# Comprehensive Analysis of ceRNA Regulation Network Involved in the Development of Coronary Artery Disease

**DOI:** 10.1155/2021/6658115

**Published:** 2021-01-14

**Authors:** Jiabei He, Xinglong Li, Yao Zhang, Qingqing Zhang, Lianhong Li

**Affiliations:** ^1^Department of Pathology and Forensic Medicine, Dalian Medical University, Dalian 116044, China; ^2^Department of Ultrasound, The Second Affiliated Hospital of Dalian Medical University, Dalian 116027, China

## Abstract

**Background:**

Coronary artery disease (CAD) is one of the most common causes of sudden death with high morbidity in recent years. This paper is aimed at exploring the early peripheral blood biomarkers of sudden death and providing a new perspective for clinical diagnosis and forensic pathology identification by integrated bioinformatics analysis.

**Methods:**

Two microarray expression profiling datasets (GSE113079 and GSE31568) were downloaded from the Gene Expression Omnibus (GEO) database, and we identified differentially expressed lncRNAs, miRNAs, and mRNAs in CAD. Gene Ontology (GO) and KEGG pathway analyses of DEmRNAs were executed. A protein-protein interaction (PPI) network was constructed, and hub genes were identified. Finally, we constructed a competitive endogenous RNA (ceRNA) regulation network among lncRNAs, miRNAs, and mRNAs. Also, the 5 miRNAs of the ceRNA network were verified by RT-PCR.

**Results:**

In total, 86 DElncRNAs, 148 DEmiRNAs, and 294 DEmRNAs were dysregulated in CAD. We received 12 GO terms and 5 pathways of DEmRNAs. 31 nodes and 78 edges were revealed in the PPI network. The top 10 genes calculated by degree method were identified as hub genes. Moreover, there were a total of 26 DElncRNAs, 5 DEmiRNAs, and 13 DEmRNAs in the ceRNA regulation network. We validated the 5 miRNAs of the ceRNA network by RT-PCR, which were consistent with the results of the microarray.

**Conclusions:**

In this paper, a CAD-specific ceRNA network was successfully constructed, contributing to the understanding of the relationship among lncRNAs, miRNAs, and mRNAs. We identified potential peripheral blood biomarkers in CAD and provided novel insights into the development and progress of CAD.

## 1. Introduction

Worldwide, coronary artery disease (CAD) is one of the most common causes of sudden death with high morbidity in recent years, severely depriving patients' quality of life and posing a heavy social-economic burden [1]. The earlier the intervention in patients with CAD is, the lesser the occurrence of sudden death. Despite the advances made in the treatment of CAD, its early diagnosis and pathogenesis remain difficult and complex, respectively. The etiology and pathogenesis of CAD are currently not well described. Therefore, the main objective of this study is to explore the mechanisms of CAD progression and seek potential new therapeutic targets for CAD treatment.

High-throughput technology has recently gained traction, providing perfect opportunities for identifying biomarkers [2]. Biomarkers and therapeutic targets of noncoding RNAs play a critical role in CAD [3]. The synergistic action of multiple genes or RNAs may result in complex diseases. The pathogenesis of CAD has received more and more attention, including epigenetic modification and ceRNA [4–6]. The mRNAs and proteins they encoded are expressed abnormally in this complex regulatory process, and the involved noncoding RNAs exhibit spatiotemporal specificity. Salmena et al. [7] presented the ceRNA hypothesis as a new pattern of regulatory mechanism for noncoding RNA and mRNA. Besides playing a critical role in the pathological process of various diseases, lncRNAs act as miRNA response elements in ceRNA [8]. There are relatively many studies on the function of miRNA in CAD. Recent research demonstrated that downregulated miRNA-26a-5p induces endothelial cell apoptosis in CAD [9]. In addition, the increased expression levels of miR-24, miR-33a, miR-103a, and miR-122 may be associated with the risk of CAD [10]. Recently, research considers lncRNAs to be noncoding RNAs involved in gene and protein expression regulation at the epigenetic level [11]. lncRNA regulates mRNA by acting as an endogenous sponge. The regulatory role of lncRNA in miRNA has attracted significant attention [12]. Nonetheless, very little information about the ceRNA network in CAD is available. A disorder of lncRNA expression, which breaks the ceRNA interaction of lncRNA/mRNA mediated by miRNA, is important in the ceRNA network. Therefore, a better understanding of the development process of the ceRNA network in CAD may contribute to a better understanding of the mechanism in CAD.

The focus of basic medical experiments is mostly cardiomyocytes or endothelial cells. Organisms respond to damages and change the blood composition during the development and progression of disease due to their interaction and dynamic nature. Peripheral blood may, therefore, be used as an effective sample for CAD analysis and as an alternative to biopsy.

In our study, two peripheral blood microarray datasets of patients with CAD and normal groups were, respectively, downloaded from GEO, including the information on the expression of lncRNA, miRNA, and mRNA. In addition, we obtained the PPI network and HUB genes. Finally, the ceRNA network of CAD was constructed through comprehensive and integration analysis. This study will help to explore potential molecular targets for new intervention strategies and provide new insights into clinical diagnosis and treatment of CAD.

## 2. Materials and Methods

### 2.1. Microarray Data

Two microarray expression profiling datasets (GSE113079 and GSE31568) were retrieved and downloaded from the Gene Expression Omnibus (GEO) database of the National Center for Biotechnology Information (NCBI) (http://www.ncbi.nlm.nih.gov/geo), a public functional genomics data repository. The GSE113079 dataset, deposited by Li L et al., was an expression profile of mRNA-lncRNA based on the GPL20115 Agilent-067406 Human CBC lncRNA+mRNA microarray V4.0. The experiment contained 141 samples of peripheral blood mononuclear cells, including 93 subjects with coronary artery disease (CAD group) and 48 healthy controls (control group). In addition, the GSE31568 dataset, deposited by Keller A et al., was a miRNA expression profile based on the GPL9040 febit *Homo Sapiens* miRBase 13.0. A total of 40 samples were collected comprising 20 samples of peripheral blood mononuclear cells from patients with coronary artery disease (CAD group) and 20 samples from healthy people (control group).

### 2.2. Data Preprocessing and Differential Expression Analysis

In addition, we downloaded the GPL20115 and GPL9040 annotation files from GEO. We transformed the probes into the respective gene symbols based on the annotation information. When several samples corresponded to the same gene, we selected the maximum expression.

GEO2R, an online analysis tool in GEO, was used to evaluate the differential expression in CAD and control groups from the two profile datasets. We defined the threshold for significant difference as ∣log_2_fold − change | >1.2 and the adjusted *P* value < 0.01. The volcano plot generated in the GraphPad Prism 8.0 software was used to show all the significant differences in mRNAs, lncRNAs, and miRNAs.

### 2.3. Gene Ontology and KEGG Pathway Enrichment Analyses

The enrichment analyses of differentially expressed mRNAs, including GO and KEGG pathway analyses, were performed using DAVID 6.8 (https://david.ncifcrf.gov/) [13–15]. Further structures in GO analysis including biological process (BP), cellular component (CC), and molecular function (MF) were performed to annotate protein functions. Statistical significance was set at *P* value < 0.05.

### 2.4. Construction of the Protein-Protein Interaction (PPI) Network and Hub Gene Identification in CAD

To construct the PPI network, the differentially expressed mRNAs were mapped into the STRING online database (https://string-db.org/) which is used to predict the protein-protein interactions (PPI) and describe the protein functional associations [16]. The interaction pair score of 0.4 was set as the threshold value. The Cytoscape software (version 3.7.1) was used to visualize and construct the PPI network intuitively [17].

In addition, the CytoHubba plugin in Cytoscape used to identify the hubba nodes was selected to measure the core genes in the PPI network [18]. The top 10 nodes were ranked by degree.

### 2.5. Construction of the lncRNA-miRNA-mRNA Competitive Endogenous RNA (ceRNA) Regulation Network in CAD

We constructed a competitive endogenous RNA regulation network among lncRNAs, miRNAs, and mRNAs based on the hypothesis that miRNAs can regulate the activity of target mRNAs, and that lncRNAs can interact with the activity of miRNAs [7]. In this hypothesis, lncRNAs act as miRNA sponges. The miRcode (http://www.mircode.org) was used to predict DElncRNA-DEmiRNA interactions [19]. In addition, we predicted the possible target DEmRNAs of DEmiRNAs by using three data online tools, Targetscan (http://www.targetscan.org) [20], miRTarBase (http://mirtarbase.mbc.nctu.edu.tw) [21], and miRDB (http://www.mirdb.org) [22]. The possible DEmiRNA-DEmRNA interactions were searched in all the three databases. Finally, we established and plotted the ceRNA network from the differentially expressed lncRNAs, miRNAs, and mRNAs by using Cytoscape software (version 3.7.1).

### 2.6. Reverse Transcription and Real-Time Quantification of miRNA Expression

A total of 20 patients were recruited from the Second Affiliated Hospital of Dalian Medical University, China. Ten subjects who had no cardiovascular disease were recruited as the control group. Ten subjects who had been diagnosed with coronary artery disease by DSA were recruited as the CAD group. Peripheral blood was collected by venipuncture in the morning. Peripheral blood mononuclear cells (PBMC) were isolated by the Ficoll reagent (Solarbio, China). Total RNA was extracted from PBMC using the TRIzol Reagent (Thermo Fisher Scientific, USA) according to the procedure supplied by the manufacturer. Reverse transcriptions were performed using EasyScript® One-Step gDNA Removal and cDNA Synthesis SuperMix (TransGen Biotech Co., Ltd., China). Real-time PCR was performed under the iCycler™ Real Time System by mixing it with the SYBR Premix EX Tag Master mixture kit (TransGen Biotech Co., Ltd., China). The reaction conditions of RT-PCR are as follows: 94°C for 30 sec and 45 cycles at 94°C for 5 sec, 60°C for 30 sec, then dissociation stage. The relative expression levels of miRNAs were calculated by the 2^−ΔΔCT^ method and normalized against U6 small nuclear RNA. The stem-loop RT and RT-PCR primers were designed, and the sequences of the primers can be found in Table 1. This research was approved by the local ethics committee, and each subject gave written informed consent.

## 3. Results

### 3.1. Identification of Differentially Expressed mRNAs, lncRNAs, and miRNAs in CAD

**Figure 1:**
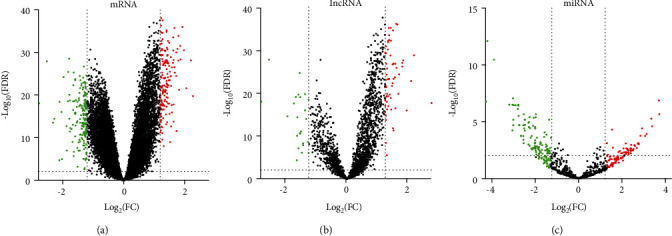
The differentially expressed mRNAs (a), lncRNAs (b), and miRNAs (c) in CAD are shown in the volcano plots mapping in GraphPad Prism 8.0 software. Different colors represent different expressions, and each spot represents a gene. Genes with no differentially expressed mRNAs, lncRNAs, and miRNAs are shown as black spots. Upregulated and downregulated genes are shown in red spots and green spots, respectively.

We used the online analysis tool GEO2R to identify the differentially expressed mRNAs and lncRNAs in peripheral blood mononuclear cells of CAD patients and healthy controls in GSE113079. In addition, we identified the differentially expressed miRNAs in GSE31568. In our findings, the GSE113079 dataset was comprised of 294 differentially expressed mRNAs (127 upregulated and 167 downregulated, Figure 1(a)) and 86 differentially expressed lncRNAs (64 upregulated and 22 downregulated, Figure 1(b)), whereas the GSE31568 dataset was comprised of 148 differentially expressed miRNAs (49 upregulated and 99 downregulated, Figure 1(c)). Tables 2–5, respectively, presents the top 20 upregulated and downregulated mRNAs, lncRNAs, and miRNAs.

### 3.2. GO and KEGG Pathway Enrichment Analyses of Differentially Expressed mRNAs in CAD

**Figure 2:**
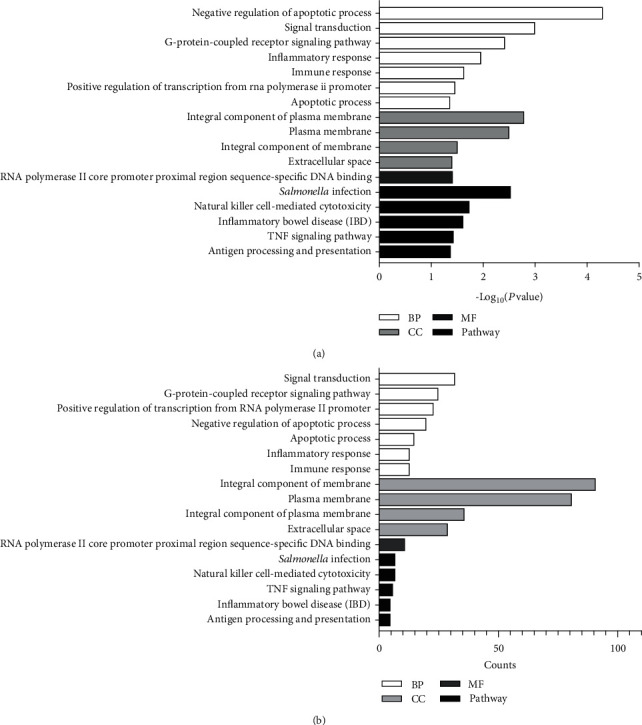
GO and KEGG pathway enrichment analyses of differentially expressed mRNAs. (a) The *y*-axis depicts the enriched functional terms and pathways. The *x*-axis represents the −log_10_(*P* value). (b) The *y*-axis depicts the enriched functional terms and pathways. The *x*-axis represents the enriched gene counts. The functional terms include the biological process (BP, in white), cellular component (CC, in light grey), and molecular function (MF, in dark grey). The enriched pathways are shown in black.

The enrichment analysis results revealed 12 GO terms and 5 pathways of differentially expressed mRNAs, including 7 biological processes (BPs), 4 cellular components (CCs), and 1 molecular function (MF). The results indicated that the differentially expressed mRNAs in BPs were mainly enriched in the negative regulation of the apoptotic process, signal transduction, G-protein-coupled receptor signaling pathway, inflammatory response, immune response, positive regulation of transcription from the RNA polymerase II promoter, and apoptotic process. The differentially expressed mRNAs in CCs were enriched primarily in the integral component of the plasma membrane, in the integral component of the membrane, and in the extracellular space. The differentially expressed mRNAs in MF were mainly enriched in RNA polymerase II core promoter proximal region sequence-specific DNA binding. In addition, we obtained the following pathways: *Salmonella* infection, natural killer cell-mediated cytotoxicity, inflammatory bowel disease (IBD), TNF signaling pathway, and antigen processing and presentation. All the results are shown in the bar graph of Figures 2(a) and 2(b) and summarized in Table 6.

### 3.3. Construction of PPI Network and Hub Gene Identification in CAD

**Figure 3:**
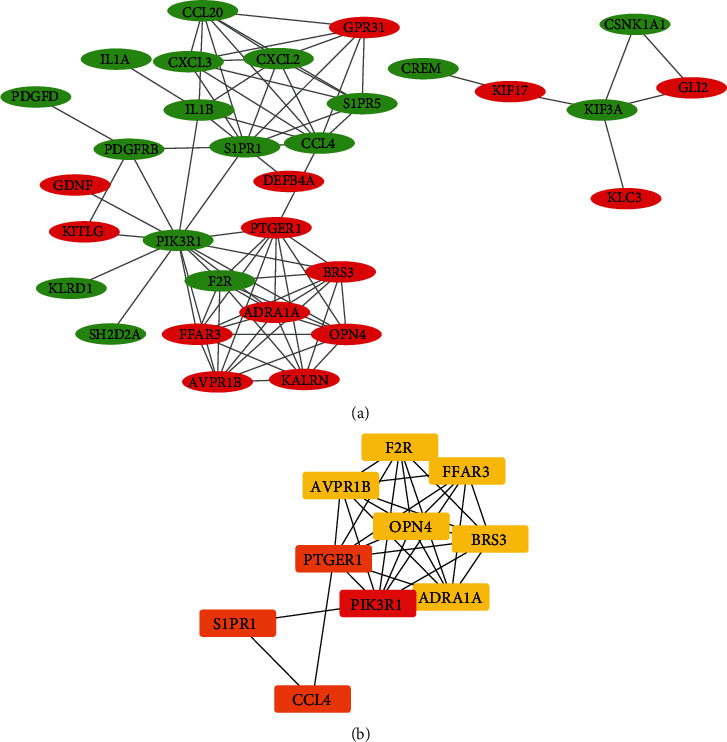
(a) PPI network of the differentially expressed mRNAs are constructed. The interaction between two genes is shown at the edge. Red represents upregulation, and green represents downregulation, respectively. (b) Hub genes in the PPI network were identified with the CytoHubba plugin ranked by degree method. The higher score has a dark red color, and the lower score has a light yellow color.

A PPI network was constructed to elucidate the interactions of the differentially expressed genes. Data analysis was based on the STRING database with the interaction pair score > 0.4 and was visualized in Cytoscape (Figure 3(a)). The PPI network revealed 31 nodes and 78 edges. There were 14 upregulated and 17 downregulated genes, and the identification of hub genes in the PPI network was done using the CytoHubba plugin in Cytoscape. The top 10 genes determined by different-color degree are shown as hub genes (Figure 3(b)). These genes contained phosphoinositide-3-kinase regulatory subunit 1 (PIK3R1), prostaglandin E receptor 1 (PTGER1), C-C motif chemokine ligand 4 (CCL4), sphingosine-1-phosphate receptor 1 (S1PR1), adrenoceptor alpha 1A (ADRA1A), arginine vasopressin receptor 1B (AVPR1B), coagulation factor II thrombin receptor (F2R), bombesin receptor subtype 3 (BRS3), free fatty acid receptor 3 (FFAR3), and opsin 4 (OPN4). The details are shown in Table 7.

### 3.4. Construction of the lncRNA-miRNA-mRNA ceRNA Regulation Network in CAD

**Figure 4:**
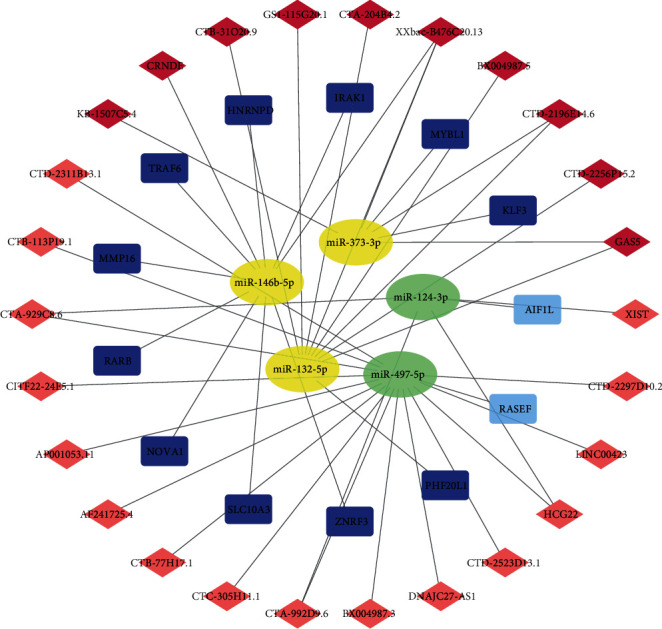
lncRNA-miRNA-mRNA ceRNA regulation network in CAD. Diamond nodes represent lncRNAs (light red=upregulation; dark red=downregulation). Rectangle nodes represent mRNAs (light blue=upregulation; dark blue=downregulation). Ellipse nodes represent miRNAs (yellow=upregulation; green=downregulation). The gray edges indicate target interactions in the network.

In the ceRNA network, there were a total of 26 lncRNAs, 5 miRNAs, and 13 mRNAs which were visualized using Cytoscape (Figure 4). First, we derived lncRNA-miRNA interactions from the miRcode database. Out of these, 44 of the 86 DElncRNAs and 26 of the 148 DEmiRNAs formed 98 lncRNA-miRNA pairs (Table 8). We only considered the interactions between miRNAs and mRNAs present in all three databases, namely, Targetscan, miRTarBase, and miRDB databases. As a result, 5 DEmRNAs and 13 DEmiRNAs formed 13 miRNA-mRNA pairs were obtained (Table 9). The information on the ceRNA network for lncRNA-miRNA-mRNA ceRNA are shown in Table 10.

### 3.5. RT-PCR Verification Results

**Figure 5:**
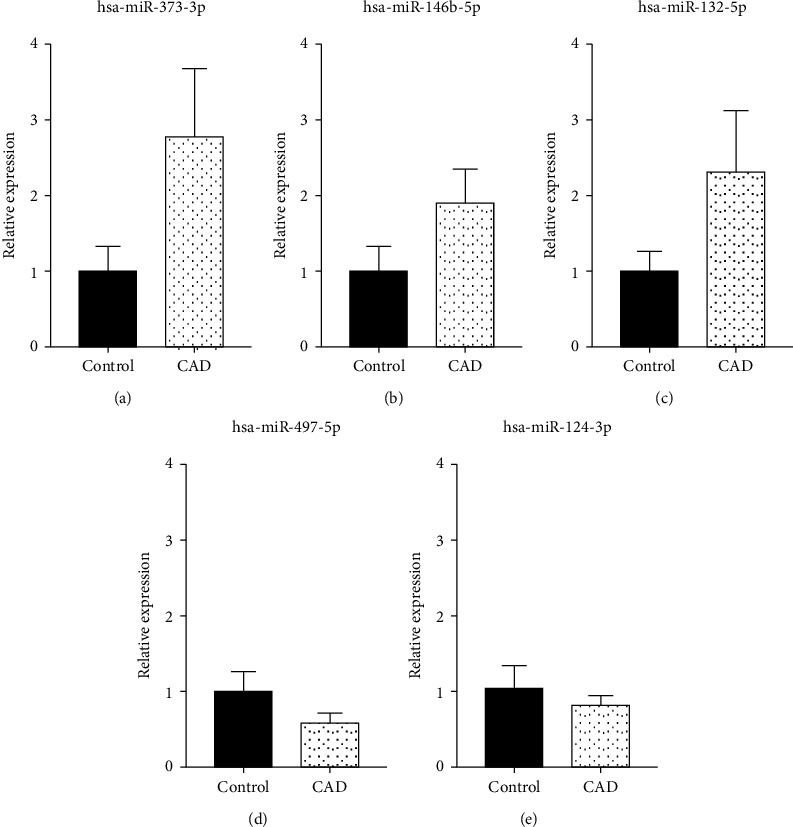
The expressions of miRNA-373-3p, miRNA-146b-5p, miRNA-132-5p, miRNA-497-5p, and miRNA-124-3p in control and CAD. Relative expression of the miRNAs by RT-PCR in 20 blood samples. (a) miR-373-3p was upregulated 2.70 times in CAD. (b) miR-146b-5p was upregulated 1.88 times in CAD. (c) miR-132-5p was upregulated 2.26 times in CAD. (d) miR-497-5p was downregulated 0.57 times in CAD. (e) miR-124-3p was downregulated 0.76 times in CAD.

The expressions of 5 miRNAs in the ceRNA network were verified with RT-PCR. Expressions of miRNA-373-3p, miRNA-146b-5p, and miRNA-132-5p in CAD were 2.70, 1.88, and 2.26 times, respectively. Expressions of miRNA-497-5p and miRNA-124-3p in CAD was 0.57 and 0.76 times, respectively (Figure 5). The trends were consistent with the results of array data.

## 4. Discussion

CAD is an international public health problem that has a significant impact on the quality of human life [1]. The most prevalent pathogenesis of sudden death is considered to be CAD, which continues to rise year after year [33]. However, the lack of biomarkers for early diagnosis of CAD poses a serious challenge in making an effective early diagnosis of CAD clinically. High-throughput biological techniques have recently been developed and are widely used in basic researches [2]. From the perspective of genetics, it can demonstrate the difference in thousands of genes in the disease development process, which may provide a biological basis for early accurate diagnosis of CAD [34, 35]. We obtained the hub genes of CAD by integrated analysis and constructed a ceRNA network which provides a foundation for further exploration of CAD progression mechanisms.

In this study, we identified a total of 294 DEmRNAs, 86 DElncRNAs, and 148 DEmiRNAs. Based on the results of the enrichment analysis, we received 12 GO terms and 5 pathways of DEmRNAs. The role is primarily linked to the apoptotic process and signal transduction. Moreover, pathways are mainly enriched in inflammation and apoptosis. In this study, we successfully constructed a ceRNA network and established the underlying mechanism between lncRNA and mRNA that can provide a new understanding of CAD therapeutic targets. Among the three types of differentially expressed RNAs in the ceRNA network, there were 26 lncRNAs, 5 miRNAs, and 13 mRNAs.

In the present study, the ceRNA network had 13 mRNAs including KLF3, MYBL1, IRAK1, HNRNPD, TRAF6, MMP16, RARB, NOVA1, SLC10A3, ZNRF3, PHF20L1, RASEF, and AIF1L. Some mRNA genes have been reported to be prominent in the ceRNA network. KLF3 belongs to the KLF family, and it is associated with adipogenesis and inflammation [36, 37]. In our present study, the expression of KLF3 was low in CAD. This result could be attributed to the epigenetic modification in CAD. This study also revealed low levels of MYBL1 in CAD. Previous reports implicated MYBL1 in adenoid cystic carcinoma and cutaneous adenocystic carcinoma [38, 39]. In addition, a high expression level of RASEF was observed in CAD. RASEF is a member of the Rab family of GTPases which are linked to the regulation of membrane traffic. Overexpression of RASEF increases P38 phosphorylation, leading to apoptosis and proliferation inhibition [40]. The expression of AIF1L, associated with calcium ion binding and actin filament binding, was found to be high in CAD. Previous studies reported low expression of AIF1L in undamaged tissues. Besides, it has been implicated in the inflammatory response associated with the rejection of human heart transplants [41].

In the present study, the ceRNA network had 5 miRNAs, including miRNA-373-3p, miRNA-146b-5p, miRNA-132-5p, miRNA-497-5p, and miRNA-124-3p. Among them, the first three (miRNA-373-3p, miRNA-146b-5p, and miRNA-132-5p) were upregulated in CAD, whereas the last two (miRNA-497-5p, miRNA-124-3p) were downregulated in CAD. Previous studies suggest that miRNA-373-3p has a strong antifibrosis effect which is significant in preventing reconstruction after infarction [42]. In previous studies, miRNA-146b-5p's expression was found to be high in atrial fibrillation in previous studies and associated with fibrosis [43]. According to the report, miRNA-132-5p may cause liver steatosis and hyperlipidemia [44]. We suspect that it might be involved in lipogenesis. Smoking reduces the ability of blood to carry oxygen, affects the role of lipid metabolism, promotes the formation of arterial plaque, and increases the incidence of CAD. The upregulation of miR-497-5p had a protective effect on human bronchial epithelial cells that had been damaged by cigarette smoke [45]. Inflammatory responses may contribute to atherosclerosis [46]. Studies have shown that miRNA-124-3p regulates the inflammatory response induced by ischemia-reperfusion injury of human myocardial cells [47].

In the present study, there were 26 lncRNAs in the ceRNA network, including 16 upregulated and 10 downregulated lncRNAs. Inflammation is not the only etiology of CAD and cannot explain the whole pathogenesis. However, it is an important factor in explaining a possible mechanism of CAD. Among the 26 lncRNAs, GAS5, CRNDE, HCG22, and XIST were associated with inflammation [48–51], while the rest have not been reported previously. GAS5 and XIST have been extensively researched in previous reports. The role of lncRNA GAS5 in atherogenesis to regulate the apoptosis of macrophages and endothelial cells through exosomes suggests that inhibition of lncRNA GAS5 can be an effective way to treat atherosclerosis [52]. GAS5 could be a successful biomarker for CAD according to the study [53]. The inhibition of XIST could improve myocardial I/R injury by regulating the miRNA-133a/SOCS2 axis and inhibiting autophagy [54]. Bioinformatics-based lncRNA analysis will be helpful for future experimental research.

While our current findings are of crucial clinical significance and may provide a basis for future studies on the mechanism of CAD, we must also pay attention to some limitations. First, based on the analysis of the databases, we should use qPCR for the verification of hub genes in clinical blood samples for further study. Secondly, our sample size of 181 was relatively small; hence, future studies should consider a larger sample size to verify the accuracy of our results. Third, the specific mechanism of the lncRNA-mRNA-miRNA network of CAD needs to be further studied in vivo and in vitro for verification.

## 5. Conclusion

In summary, we have constructed a CAD-specific ceRNA network to aid in further understanding the relationship among lncRNAs, miRNAs, and mRNAs. Furthermore, we identify potential therapeutic targets through public database integrated analysis and provided novel insights into the development and progress of CAD.

## Figures and Tables

**Table 1:** RT-PCR primer sequences.

Gene name		Sequences 5′-3′
hsa-miR-373-3p	Stem-loop primer	GTCGTATCCAGTGCAGGGTCCGAGGTATTCGCACTGGATACGACacaccc
	Forward primer	GAAGTGCTTCGATTTTG
	Reverse primer	CCAGTGCAGGGTCCGAGGT

hsa-miR-146b-5p	Stem-loop primer	GTCGTATCCAGTGCAGGGTCCGAGGTATTCGCACTGGATACGACaccaga
	Forward primer	GCCCTGTGGACTCAGT
	Reverse primer	CCAGTGCAGGGTCCGAGGT

hsa-miR-132-5p	Stem-loop primer	GTCGTATCCAGTGCAGGGTCCGAGGTATTCGCACTGGATACGACagtaac
	Forward primer	ACCGTGGCTTTCGATT
	Reverse primer	CCAGTGCAGGGTCCGAGGT

hsa-miR-497-5p	Stem-loop primer	GTCGTATCCAGTGCAGGGTCCGAGGTATTCGCACTGGATACGACacaaac
	Forward primer	CAGCAGCACACTGTG
	Reverse primer	CCAGTGCAGGGTCCGAGGT

hsa-miR-124-3p	Stem-loop primer	GTCGTATCCAGTGCAGGGTCCGAGGTATTCGCACTGGATACGACttggca
	Forward primer	TAAGGCACGCGGTGAA
	Reverse primer	CCAGTGCAGGGTCCGAGGT

U6	Stem-loop primer	GGAACGCTTCACGAATTTGC
	Forward primer	CGCTTCGGCAGCACATATACTA
	Reverse primer	GGAACGCTTCACGAATTTGC

**Table 2:** Top 20 upregulated mRNAs and lncRNAs.

mRNA			lncRNA		
mRNA name	LogFC	FDR	lncRNA name	LogFC	FDR
ACTBL2	2.06	2.52*E*‐22	CTD-2306M5.1	2.65	2.36*E*‐18
BIRC7	1.98	2.99*E*‐31	CTD-2311B13.1	2.1	1.95*E*‐29
KIF17	1.93	1.04*E*‐36	CTB-113P19.1	2.01	1.91*E*‐23
NMNAT2	1.87	4.59*E*‐29	STARD4-AS1	1.87	1.28*E*‐16
NEURL1B	1.86	3.11*E*‐32	CTD-2012 M11.3	1.76	2.75*E*‐28
OPN4	1.8	1.31*E*‐34	CTD-3116E22.7	1.73	8.57*E*‐27
FTMT	1.8	8.56*E*‐29	TPRG1-AS1	1.73	1.13*E*‐27
TRPM5	1.77	1.51*E*‐31	CTA-929C8.6	1.63	5.34*E*‐25
NUPR1	1.73	1.03*E*‐30	CERS6-AS1	1.61	1.76*E*‐20
AVPR1B	1.73	3.24*E*‐25	AP000439.3	1.58	1.33*E*‐36
PYDC2	1.68	5.65*E*‐26	TDRG1	1.55	1.68*E*‐26
SHANK1	1.67	1.99*E*‐36	LINC00423	1.54	9.02*E*‐37
PLA2R1	1.67	2.94*E*‐27	CTD-2297D10.2	1.52	4.07*E*‐17
TREH	1.64	1.80*E*‐14	CTD-2313P7.1	1.52	1.82*E*‐27
CPEB1	1.64	6.15*E*‐29	LINC00511	1.5	2.53*E*‐24
OR4C3	1.63	3.21*E*‐31	CITF22-24E5.1	1.46	5.94*E*‐24
TM4SF5	1.62	2.88*E*‐28	DISC1FP1	1.46	6.09*E*‐12
EYS	1.59	3.30*E*‐27	LINC00283	1.46	1.65*E*‐27
MYL2	1.59	1.25*E*‐16	AP001053.11	1.45	1.77*E*‐27
MYF5	1.58	2.43*E*‐18	DMD-AS3	1.44	1.74*E*‐12

**Table 3:** Top 20 downregulated mRNAs and lncRNAs.

mRNA			lncRNA		
mRNA name	LogFC	FDR	lncRNA name	LogFC	FDR
ARHGEF33	-2.78	8.46*E*‐19	LA16c-390E6.5	-1.86	3.03*E*‐15
PDE4D	-2.52	1.30*E*‐28	GAS5	-1.66	2.93*E*‐18
RBPJL	-2.32	3.12*E*‐14	MED14-AS1	-1.6	9.42*E*‐20
PAK2	-2.28	3.94*E*‐15	KB-1507C5.4	-1.57	5.15*E*‐05
MTRNR2L9	-2.11	2.11*E*‐05	CTD-2017C7.2	-1.56	1.04*E*‐10
C22orf31	-2.1	3.88*E*‐19	AE000661.37	-1.55	5.34*E*‐15
CCL4	-2.04	9.51*E*‐17	CTD-2308G16.1	-1.52	8.88*E*‐08
IL1A	-2.03	1.08*E*‐05	TEX26-AS1	-1.52	2.67*E*‐20
CSNK1A1	-1.97	5.96*E*‐25	CTD-2256P15.2	-1.48	2.50*E*‐25
AREG	-1.87	1.05*E*‐07	CTD-2196E14.6	-1.47	2.57*E*‐11
KIR2DS4	-1.83	2.79*E*‐19	CRNDE	-1.46	3.38*E*‐11
AKAP5	-1.82	3.83*E*‐26	GS1-115G20.1	-1.46	1.06*E*‐19
FAM169A	-1.79	3.02*E*‐29	CTD-2014E2.6	-1.44	1.02*E*‐11
WHSC2	-1.78	3.57*E*‐15	BX004987.5	-1.41	9.05*E*‐07
BNC2	-1.78	8.77*E*‐21	AF213884.2	-1.37	1.58*E*‐08
SH2D4A	-1.76	3.94*E*‐12	XXbac-B476C20.13	-1.37	6.15*E*‐09
FAM47A	-1.76	1.83*E*‐19	AL162151.4	-1.31	2.04*E*‐20
LONRF3	-1.75	5.84*E*‐13	CTA-204B4.2	-1.31	1.22*E*‐16
KRT33B	-1.71	9.94*E*‐13	CTB-31O20.9	-1.27	1.25*E*‐14
C9orf170	-1.68	1.05*E*‐09	UBOX5-AS1	-1.22	2.80*E*‐09

**Table 4:** Top 20 upregulated miRNAs.

miRNA name	LogFC	FDR
hsa-miR-302b	3.6444	2.19*E*‐06
hsa-miR-1290	3.62242	1.34*E*‐07
hsa-miR-508-3p	3.32377	2.58*E*‐05
hsa-miR-126	3.28998	5.43*E*‐06
hsa-miR-1258	3.0678	1.22*E*‐04
hsa-miR-609	3.00346	1.81*E*‐04
hsa-miR-302d	2.86326	3.96*E*‐05
hsa-miR-1468	2.7974	1.74*E*‐04
hsa-miR-190b	2.72287	9.60*E*‐04
hsa-miR-520c-3p	2.70583	7.77*E*‐04
hsa-miR-548j	2.68311	8.96*E*‐04
hsa-miR-204	2.67914	8.27*E*‐04
hsa-miR-376a	2.63529	2.06*E*‐03
hsa-miR-375	2.55779	1.45*E*‐03
hsa-miR-135a	2.52481	1.13*E*‐03
hsa-let-7b	2.47967	2.53*E*‐03
hsa-miR-142-3p	2.46877	3.21*E*‐03
hsa-miR-942	2.4338	3.37*E*‐03
hsa-miR-382	2.40268	3.37*E*‐03
hsa-miR-455-5p	2.37485	1.10*E*‐03

**Table 5:** Top 20 downregulated miRNAs.

miRNA name	LogFC	FDR
hsa-miR-1283	-4.10131	8.15*E*‐13
hsa-miR-519e	-3.10991	3.21*E*‐07
hsa-miR-518a-3p	-2.97144	3.21*E*‐07
hsa-miR-1201	-2.97091	5.84*E*‐05
hsa-miR-31	-2.94672	8.67*E*‐08
hsa-miR-491-3p	-2.92979	2.04*E*‐05
hsa-miR-10b	-2.92882	6.77*E*‐06
hsa-miR-566	-2.91617	3.59*E*‐07
hsa-miR-488	-2.82797	3.59*E*‐07
hsa-miR-1278	-2.72815	7.56*E*‐07
hsa-miR-515-5p	-2.70448	3.90*E*‐07
hsa-miR-1291	-2.70428	3.21*E*‐07
hsa-miR-520d-5p	-2.69493	3.08*E*‐06
hsa-miR-568	-2.67044	2.10*E*‐05
hsa-miR-298	-2.6596	2.04*E*‐05
hsa-miR-802	-2.58926	1.81*E*‐04
hsa-miR-616	-2.58413	1.74*E*‐04
hsa-miR-1245	-2.48371	1.85*E*‐04
hsa-miR-516a-5p	-2.48146	2.04*E*‐05
hsa-miR-520h	-2.4776	3.88*E*‐04

**Table 6:** Significantly enriched GO terms and KEGG pathways in mRNAs of DEGs.

Category	Term	Description	Count	*P* value
BP	GO:0007165	Signal transduction	32	9.88*E*‐04
BP	GO:0007186	G-protein-coupled receptor signaling pathway	25	3.73*E*‐03
BP	GO:0045944	Positive regulation of transcription from RNA polymerase II promoter	23	3.37*E*‐02
BP	GO:0043066	Negative regulation of apoptotic process	20	4.86*E*‐05
BP	GO:0006915	Apoptotic process	15	4.24*E*‐02
BP	GO:0006954	Inflammatory response	13	1.08*E*‐02
BP	GO:0006955	Immune response	13	2.28*E*‐02
CC	GO:0016021	Integral component of membrane	91	3.05*E*‐02
CC	GO:0005886	Plasma membrane	81	3.07*E*‐03
CC	GO:0005887	Integral component of plasma membrane	36	1.59*E*‐03
CC	GO:0005615	Extracellular space	29	3.87*E*‐02
MF	GO:0000978	RNA polymerase II core promoter proximal region sequence-specific DNA binding	11	3.78*E*‐02
Pathway	hsa05132	*Salmonella* infection	7	2.89*E*‐03
Pathway	hsa04650	Natural killer cell-mediated cytotoxicity	7	1.81*E*‐02
Pathway	hsa05321	Inflammatory bowel disease (IBD)	5	2.37*E*‐02
Pathway	hsa04668	TNF signaling pathway	6	3.64*E*‐02
Pathway	hsa04612	Antigen processing and presentation	5	4.11*E*‐02

**Table 7:** Top 10 in network ranked by degree method.

Gene symbol	Full name	Score	Implications
PIK3R1	Phosphoinositide-3-kinase regulatory subunit 1	14	PIK3R1 plays a central role in suppressing platelet aggregation [23]
PTGER1	Prostaglandin E receptor 1	9	PTGER1 is one of four receptors identified for PGE2. Hypoxia regulates PGE2 release and promotes PTGER1 expression [24]
CCL4	C-C motif chemokine ligand 4	9	The inflammatory microenvironment influences cell recruitment and activation [25]
S1PR1	Sphingosine-1-phosphate receptor 1	9	The loss of S1PR1 exacerbates post-MI cardiac remodeling and worsened cardiac dysfunction [26]
ADRA1A	Adrenoceptor alpha 1A	8	ADRA1A prevent a maladaptive cardiac response to pressure overload [27]
AVPR1B	Arginine vasopressin receptor 1B	8	AVPR1B appears to play the most important role in regulating erythropoiesis [28]
F2R	Coagulation factor II thrombin receptor	8	F2R can significantly contribute to the risk of ischemic events [29]
BRS3	Bombesin receptor subtype 3	8	BRS3 plays an important role in glucose/energy homeostasis [30]
FFAR3	Free fatty acid receptor 3	8	FFAR3 induces the H9C2 cells apoptosis during ischemic hypoxia and reoxygenation [31]
OPN4	Opsin 4	8	OPN4 can effectively induce significant vasorelaxation subjected to acute hypoxia [32]

**Table 8:** The interactions between differentially expressed lncRNAs and miRNAs in the miRcode database.

miRNA	lncRNA
miR-518a-3p	CTD-2306M5.1, TPRG1-AS1, CTA-929C8.6, CTB-43E15.4, H19, C20orf166-AS1, TDRG1
miR-497-5p	CTD-2311B13.1, CTB-113P19.1, CTA-929C8.6, CITF22-24E5.1, AP001053.11, AF241725.4, CTB-77H17.1, CTC-305H11.1, CTA-992D9.6, BX004987.3, DNAJC27-AS1, CTD-2523D13.1, HCG22, LINC00423, CTD-2297D10.2
miR-489	HTR4-IT1, CTA-992D9.6, BX004987.3
miR-363	TPRG1-AS1, AP001053.11, CTA-992D9.6, BX004987.3, XIST, CTC-359M8.1
miR-31	CTA-929C8.6, CTD-2281E23.3
miR-21	CTD-2139B15.2, XIST, CTD-2127H9.1, CTC-366B18.2
miR-208a	CTB-113P19.1
miR-200a	CTC-359M8.1
miR-192	DMD-AS3, BX004987.3, DNAJC27-AS1, XIST, CTC-359M8.1, TDRG1
miR-182	CTA-929C8.6
miR-155	PEX5L-AS1
miR-145	CTA-929C8.6, BX004987.3, LINC00113
miR-139-5p	CTA-992D9.6, CTC-359M8.1
miR-1297	HTR4-IT1, DNAJC27-AS1, XIST, PEX5L-AS1, CTC-359M8.1
miR-124-3p	CTA-929C8.6, CTA-992D9.6, XIST, HCG22
miR-132-5p	GAS5, CTD-2256P15.2, CTD-2196E14.6, BX004987.5, XXbac-B476C20.13, CTA-204B4.2, GS1-115G20.1, CTB-31O20.9
miR-142-3p	CTD-2308G16.1, CTD-2196E14.6, CTA-204B4.2, CRNDE
miR-146b-5p	XXbac-B476C20.13, CRNDE
miR-203	AE000661.37, BX004987.5, XXbac-B476C20.13, CRNDE
miR-204	BX004987.5, CTD-2341M24.1
miR-373-3p	GAS5, KB-1507C5.4, CTD-2196E14.6, XXbac-B476C20.13
miR-375	AE000661.37
miR-455-5p	GAS5, BX004987.5, XXbac-B476C20.13
miR-508-3p	BX004987.5, XXbac-B476C20.13, CRNDE
miR-613	KB-1507C5.4, AE000661.37, BX004987.5
miR-876-3p	AE000661.37, BX004987.5, CRNDE

**Table 9:** The interactions between differentially expressed miRNAs and mRNAs in the Targetscan, miRTarBase, and miRDB databases.

miRNA	mRNA
miR-373-3p	KLF3, MYBL1
miR-146b-5p	IRAK1, HNRNPD, TRAF6, MMP16, RARB, NOVA1, SLC10A3, ZNRF3
miR-132-5p	PHF20L1
miR-497-5p	RASEF
miR-124-3p	AIF1L

**Table 10:** The lncRNA-miRNA-mRNA ceRNA regulation network.

miRNA	mRNA	lncRNA
miR-373-3p	KLF3, MYBL1	GAS5, KB-1507C5.4, CTD-2196E14.6, XXbac-B476C20.13
miR-146b-5p	IRAK1, HNRNPD, TRAF6, MMP16, RARB, NOVA1, SLC10A3, ZNRF3	XXbac-B476C20.13, CRNDE
miR-132-5p	PHF20L1	GAS5, CTD-2256P15.2, CTD-2196E14.6, BX004987.5, XXbac-B476C20.13, CTA-204B4.2, GS1-115G20.1, CTB-31O20.9
miR-497-5p	RASEF	CTD-2311B13.1, CTB-113P19.1, CTA-929C8.6, CITF22-24E5.1, AP001053.11, AF241725.4, CTB-77H17.1, CTC-305H11.1, CTA-992D9.6, BX004987.3, DNAJC27-AS1, CTD-2523D13.1, HCG22, LINC00423, CTD-2297D10.2
miR-124-3p	AIF1L	CTA-929C8.6, CTA-992D9.6, XIST, HCG22

## Data Availability

Two microarray expression profiling datasets (GSE113079 and GSE31568) were retrieved and downloaded from the Gene Expression Omnibus (GEO) database of the National Center for Biotechnology Information (NCBI) (http://www.ncbi.nlm.nih.gov/geo).
